# Association Between Mediterranean Diet Consumption and the Physical and Mental Components of HRQL in Community-Dwelling Older Adults in Valencia

**DOI:** 10.3390/nu17203243

**Published:** 2025-10-15

**Authors:** Pilar Pérez-Ros, Ayde Rios-Corral, Omar Cauli

**Affiliations:** 1 Department of Nursing, University of Valencia, 46010 Valencia, Spain; maria.p.perez-ros@uv.es (P.P.-R.); ayde.rios@uv.es (A.R.-C.); 2 Chair of Healthy, Active and Participative Ageing, University of Valencia, 46010 Valencia, Spain; 3 Frailty and Cognitive Impairment Group (FROG), University of Valencia, 46010 Valencia, Spain

**Keywords:** quality of life, fish, nutrition, older adults, fruit, mental health

## Abstract

**Background/Objectives:** The relationship between health-related quality of life (HRQL) in community-dwelling older adults and the consumption of foods typical of the Mediterranean diet (MD) has not been studied. The main objective of this study was to determine which MD foods, as well as overall MD adherence, are associated with HRQL in community-dwelling older adults, taking into account both physical and mental components. **Methods:** A cross-sectional study was conducted including community-dwelling older adults aged 60 years or over. Sociodemographic variables, the Spanish version of the SF-12v2 quality-of-life questionnaire (physical and mental component scores), and data on the consumption of MD foods and adherence to the Mediterranean Diet Adherence Screener (MEDAS) were collected. Two binary logistic regression models, adjusted for age and sex, were fitted to analyse which food types included in the MEDAS questionnaire were significantly associated with a higher probability of having good physical and mental components of HRQL. **Results:** A total of 285 participants were recruited, with a mean age of 74.97 (SD 5.75) years, predominantly female (87.7%, *n* = 250) and 36.3% (*n* = 104) lived alone. The sample showed low physical quality of life [PCS-12: 42.88 IQR (33.61–51.09)], moderately good mental quality of life [MCS-12: 51.09 (39.97–57.42)] and good adherence to the Mediterranean diet [MEDAS: 9 (8–10)]. Binary logistic regression for PCS-12 showed that younger age, the joint consumption of less than one serving of butter per day, less than one cup of sugar-sweetened beverages per day and two or more servings of vegetables per day were significantly (*p* < 0.05) associated with good physical quality of life. Similarly, living alone, four or more tablespoons of olive oil per day and less than 2 servings of desserts per week were associated with good mental quality of life (MCS-12). **Conclusions:** The Mediterranean diet is related to physical and mental quality of life in older adults, with the consumption of specific foods within the MD showing significant associations in multivariate analyses. Identifying the items that are most closely related to good physical and mental health is key to promoting healthy lifestyle habits that are directly linked to improving quality of life from a holistic perspective. Understanding the associations between quality of life and consumption or avoidance of certain foods could help inform future nutritional interventions aimed at improving both physical and mental health in older adults.

## 1. Introduction

One of the most significant demographic changes attracting attention is the ageing of the world’s population, which is even more pronounced in Western countries [1]. Economic and social development, medical advances, declining fertility rates, and increasing life expectancy have led to substantial changes in the structure of the world’s population in recent decades [2]. Moreover, nowadays it is not only about prolonging life, but also about quality of life (QOL) and how it is experienced. Today, the emphasis is on dynamic ageing, which means that as the older population increases, their quality of life, including HRQL, must also be taken into account [3,4]. Due to the increase in the older population, it is necessary to explore and address factors associated with better quality of life to promote the health of these individuals and prevent or reduce their disease burden [5,6,7,8]. In older populations, higher HRQL scores appear to be linked to better nutritional status [9,10,11] and improved overall health [10,12,13,14]. The paucity of studies over the last two decades suggests that research on nutrition and HRQL among community-dwelling older adults remains underdeveloped [15,16].

In the Mediterranean basin region, a widely recognised healthy diet is represented by the Mediterranean diet (MD). It is characterised by high consumption of fruits, vegetables, legumes, and cereals, with fish and olive oil as the main source of cooking fat [17,18,19]. The recently revised MD pyramid emphasizes that it accounts for not just nutrition, but also sustainability, environmental concerns, economic factors, and sociocultural aspects. It represents the collaborative work of several experts who have refreshed the pyramid in light of the current challenges faced by Mediterranean countries [20,21]. In comparison to the earlier version, the last update places a greater emphasis on reducing the intake of red meat and dairy products from cattle, while encouraging an increased consumption of legumes and locally sourced, environmentally friendly plant-based foods whenever feasible [21].

Results from the SUN project, a longitudinal cohort study of 11,015 individuals in Spain [22], showed a linear association between adherence to the Mediterranean diet and the physical domains related to HRQL. Similarly, a cross-sectional study of Italian participants from the Molisani Project, a population-based cohort study, reported that adherence to a Mediterranean diet pattern was associated with better HRQL quality [23]. Furthermore, a population-based study conducted in Spain with a random sample of individuals aged between 35 and 74 years and estimating MD adherence using a score based on the tertile distribution of energy-adjusted food consumption characteristic of the Mediterranean region, also reported that good MD adherence was associated with better HRQL [24,25]. Among older Greek adults, high MD adherence was associated with better quality of life [23]. Some studies report that the association between MD adherence and HRQL is stronger for the mental health component than the physical health component [21], although other studies do not support this finding [22]. However, this observation has not always been reported [26]. In addition, there is a paucity of research investigating the relationship between the consumption of specific foods and quality of life (fish [27,28], fruits and vegetables [29,30,31,32,33], nuts [30]), and even fewer studies have examined the physical and mental components of HRQL separately in Mediterranean countries. The role of consumption of healthy specific foods in HRQL has been suggested in recent studies. For instance, increased intake of specific animal-based foods (meat, poultry, eggs, milk, and fish) was associated with improved physical HRQL after adjusting for all covariates in a population-based study of nearly 40,000 individuals conducted in China [34]. However, regarding the mental component of HRQL, positive associations were only observed for seafood consumption among men and egg consumption among women [33]. Consumption of meat, eggs and dairy products has also been associated with better mental health in Chinese older adults [33]. In the Iranian population, higher intake of ultra-processed foods was associated with poorer physical health [35].

Therefore, the main objective of this study was to determine which foods of the MD are associated with health-related quality of life in its physical and mental components in community-dwelling older adults, and to assess whether good adherence to the MD is associated with these two components of HRQL.

## 2. Materials and Methods

### 2.1. Study Design and Population

A cross-sectional study was conducted between September 2024 and April 2025. The study population comprised older adults aged 60 years or over residing in the city of Valencia, Spain, who attended the Valencia Municipal Activity Centres for Older Adults administered by the Department of Active Ageing of the Valencia City Council. This research was carried out in accordance with the requirements of the Declaration of Helsinki, and the entire study protocol was approved by the Ethics Committee of the University of Valencia, Spain (approval reference 1577979, 4 March 2021).

### 2.2. Study Sample

The study population comprised older adults aged 60 years or over residing in the city of Valencia, Spain, who attended the Valencia Municipal Activity Centres for Older Adults administered by the Department of Active Ageing of the Valencia City Council. In these centers and in this type of activities (attendance at cognitive stimulation workshops and physical exercise-based activities) the women participated much more than men as previously reported [36,37,38]. This has been replicated in different studies from our group with the framework of the Chair of Active Ageing initiatives aimed to improve quality of life in community-dwelling older individuals (https://www.uv.es/uvweb/institucional-chairs/en/list-institutional-chairs/chair-healthy-active-participatory-ageing/presentation-1286001017721.html (accessed on 10 September 2025)). To calculate the required sample size, we hypothesised, a priori, a moderate association (r = 0.4) between adherence to MD and quality of life score. A two-tailed test, α = 0.05, 95% confidence interval (95% CI), β = 0.20, and power of (1 − β) = 0.80 were also assumed. Anticipating a 20% dropout rate due to incomplete questionnaires, the required sample size was 59. This estimate was based on the classification of coefficients as weak (<0.3), moderate (0.3–0.7), or strong (> 0.7) [39,40], and application of the ARCSINUS approximation [41].

### 2.3. Information Collection Procedure

For data collection, an assessment instrument consisting of sociodemographic variables (age, sex), cohabitation (alone or with one or more partners), the Spanish version of the SF-12v2 quality-of-life questionnaire [42], and the Mediterranean Diet Adherence Screener (MEDAS) [43] was administered to evaluate quality of life and adherence to the Mediterranean diet. These instruments have been previously validated in Spanish [44,45,46,47]. For data collection purposes, an assessment tool (in paper format) was administered as an anonymous questionnaire to older adults participating in workshops at Activity Centers for Older Adults managed by the Active Aging Department of the Valencia City Council. Seniors filled by their own the questionnaire, and the participation was voluntary and unpaid.

### 2.4. Quality of Life

The SF-12v2 questionnaire generates Physical Component Summary (PCS-12) and Mental Component Summary (MCS-12) scores, each ranging from 0 to 100, with higher scores indicating better health. These scores are derived from responses to 12 questions covering physical and mental health domains. Version 2 of the SF-12 was released in 2002 [48]. The two biggest changes were a decrease in response options for the Mental Health and Vitality dimensions from 6 to 5 and an increase in response options from 2 to 5 for the Role, Physical, and Mental items to lessen the high ceiling effects they presented. The format and language were also modified to improve readability, decrease missing values, and improve compatibility with other culturally adapted versions [42,48]. The PCS-12 focuses on physical functioning, pain, and general health, while the MCS-12 assesses mental health, including emotional well-being and social functioning. PCS-12 (Physical Score) ranges from 0 to 100, with higher scores indicating better physical health and scores of 50 or lower indicating low physical health. MCS-12 (Mental Score) ranges from 0 to 100, with higher scores indicating better mental health and scores of 42 or lower indicating low mental health.

### 2.5. Mediterranean Diet Adherence Questionnaire

Adherence to the Mediterranean diet, recognized as a healthy dietary pattern in the Mediterranean region, was assessed using the specific 14-item questionnaire (MEDAS), developed by the Prevention with Mediterranean Diet (PREDIMED) group to assess adherence to the Mediterranean diet. Depending on whether participants adhere to each 14 MEDAS component (1 point) or not (0 points) (Table 1).

The adherence score ranged from 0 to 14, with higher scores indicating better adherence. Cut-off scores were defined as follows: weak adherence, 0–5 points; moderate to fair adherence, 6–9 points; and good adherence, 10 or more points [47].

### 2.6. Statistical Analyses

The variables were reported as proportions and/or means and standard deviations (SD), or medians with interquartile ranges (IQR). The Shapiro–Wilk test was used to assess normality, and no significant outliers were detected by visual inspection of boxplot. Nonparametric tests (Mann–Whitney U and Kruskal–Wallis) were used to compare median PCS-12 and MCS-12 scores. To assess the robustness of the findings against Type I errors due to multiple comparisons, a sensitivity analysis was conducted using the False Discovery Rate (FDR) adjustment via the Benjamini–Hochberg method. This procedure was applied to the *p* values obtained from two sets of analyses (PCS-12 and MECS-12), corresponding to different items from the MEDAS questionnaire. The FDR adjustment controls the expected proportion of false positives among the results deemed statistically significant. A significance threshold of q = 0.05 was used. For each *p* value, its rank, critical value, and adjusted significance status were calculated. The adjusted results are presented in the supplementary file, indicating which items remained statistically significant after controlling for FDR. This approach provides a more conservative and reliable assessment of statistical significance in the context of multiple testing (Table S1).

Two binary logistic regression models, adjusted for age and sex, were fitted for good physical and mental health to investigate the significance of MEDAS items. Variables included in the backward Wald model were age as a continuous variable, sex (male vs. female), cohabitation (alone vs. with one or more partner) and MEDAS items reporting odds ratios, 95% CIs, overall model χ^2^, Nagelkerke’s R^2^, measures of discrimination (AUC/ROC) and calibration (Hosmer–Lemeshow) and sensitivity and specificity for each model feasible, including a brief calibration plot in the Supplement (Figures S1 and S2). Indeed, we provide a short sensitivity analysis modelling PCS-12 and MCS-12 as continuous outcomes using robust linear regression.

The study data were entered into Microsoft Excel spreadsheets and analysed using SPSS Statistics (Version 28.0. Armonk, NY, USA: IBM Corp.).

## 3. Results

### 3.1. Characteristics of the Sample

A total of 285 participants were recruited, with a mean age of 74.97 (SD 5.75) years, ranging from 60 to 93 years, and predominantly female (87.7%, *n* = 250). Most participants lived with at least one other person, while 36.3% (*n* = 104) lived alone. The participants belong to 16 different centers, and the participation rate was 98%. The sample showed median scores indicating low physical quality of life, good mental quality of life and suboptimal adherence to the Mediterranean diet (Table 2).

### 3.2. Relationship Between Quality of Life, Sociodemographic Factors, and Adherence to the Mediterranean Diet

The quality-of-life score assessed using the SF-12 scale indicated a low quality of life in the PCS-12 physical dimension, with a median score below 50 points; 28.8% of participants (*n* = 82) reported good physical quality of life. Physical health did not differ according to sex, cohabitation, or age group, with participants aged 80 years and over having the lowest physical quality of life (37.7 points). Meanwhile, the mental dimension (MCS-12) showed a median slightly above 50, with 70.9% of participants (*n* = 202) reporting good mental quality of life (Table 2 and Table 3). Differences were found in mental health by sex, with women having the lowest quality of life (50.65 points vs. 55.96 in men; *p* = 0.009) (Table 3). No significant differences were found in the mental dimension according to age groups or cohabitation. No differences were found in Mediterranean diet adherence scores according to sex, age group, or cohabitation status (Table 3).

**Figure 1:**
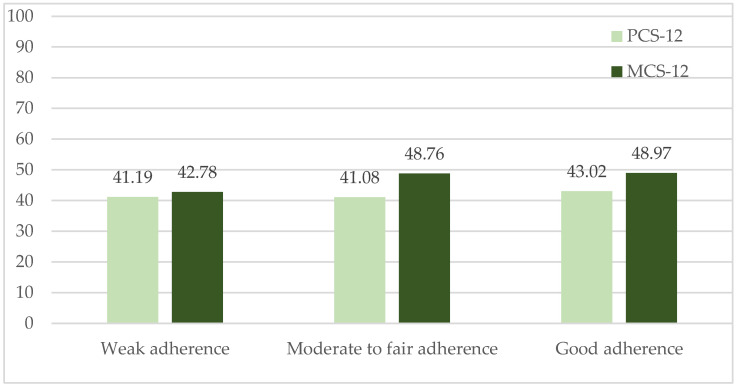
Quality-of-life scores (SF-12) in physical (PCS-12) and mental (MCS-12) dimensions according to categories of Mediterranean diet adherence (MEDAS). Legend: PCS-12 (Physical Score) 0–100 points with higher scores indicating better physical health; MCS-12 (Mental Score) 0–100 points, with higher scores indicating better mental health. MEDAS: Mediterranean Diet Adherence. Weak adherence, 0–5 points; moderate to fair adherence, 6–9 points; good adherence, 10–14 points.

No differences were found in the quality-of-life scores for either dimension (PCS-12, *p* = 0.336; MCS-12, *p* = 0.166) according to the categories of good (*n* = 106), moderate (*n* = 167), and poor adherence (*n* = 12) to the Mediterranean diet (Figure 1).

In order to explore the possible implications of the Mediterranean diet on quality of life, we analysed the differences between each of the items on the MEDAS scale. Physical quality of life (PCS-12) was lower among individuals who consumed more saturated fats than recommended, specifically, butter (<1 serving: 43.12 vs. 36.59; *p* = 0.008) and desserts (<2 servings: 43.53 vs. 39.86; *p* = 0.034). In the mental dimension (MCS-12), a lower quality of life was observed in those who did not meet the criterion of daily olive oil consumption (≥4 servings: 53.73 vs. 49.32, *p* = 0.032). Conversely, a higher score was found in participants who did not meet the criterion of daily fruits (≥3 servings: 49.44 vs. 53.09; *p* = 0.027) and weekly fish consumption (≥3 servings: 48.98 vs. 52.95; *p* = 0.029). Only four people consumed seven or more servings of wine per week, including two women and two men. (Table 4). Following the FDR adjustment, only a subset of items retained statistical significance. Specifically, in the PCS-12 analysis, the item “Butter < 1” remained significant, while in the MCS-12 analysis, the items “Fruits ≥ 3” and “Fish ≥ 3” were statistically significant after correction. These findings suggest that the associations observed for these dietary components are robust and less likely to be false positives due to multiple testing (Table S1).

### 3.3. Factors Related to Low Quality of Life in the Physical and Mental Dimensions

To quantify the association between the sociodemographic variables and good physical and mental quality of life, two binary logistic regressions were performed. The PCS-12 logistic regression model was statistically significant (Chi^2^ = 36.09; *p* < 0.001; Nagelkerke’s R^2 =^ 0.173) with adequate calibration (Hosmer and Lemeshow test; Chi^2^ = 11.23; *p* = 0.189, Figure S1) and correctly classified 70.1% of the cases, with a sensitivity of 15.9% and a specificity of 93.1%. The AUC is 0.704. (95%CI:0.642–0.765; *p* < 0.001) with *n* = 82 positive cases.

Consuming two or more servings of vegetables per day (OR = 1.89; 95% CI: 0.97–3.68; defined one serving: 200 g [consider side dishes as half a serving]), consuming less than one serving of butter, margarine, or cream per day (OR = 12.23; 95% CI: 1.58–94.93, (1 serving: 12 g) and those consuming less than one cup (1 cup = 100 mL) of sugar-sweetened beverages per day (OR = 4.19; 95% CI: 1.19–14.90) were more likely to report good physical quality of life (Table 5). On the other hand, age (OR = 0.91; 95% CI: 0.86–0.97) and preference for white meat instead of red meat (OR = 0.46; 95% CI: 0.20–1.07) reported lower physical condition.

The MCS-12 logistic regression model was statistically significant (Chi^2^ = 18.80; *p* < 0.001; Nagelkerke’s R^2 =^ 0.095) with adequate calibration (Hosmer and Lemeshow test; Chi2 = 13.52; *p* = 0.259; Figure S2) and correctly classified 73% of the cases, with a sensitivity of 96.5% and a specificity of 15.7%. The AUC is 0.646. (95%CI:0.575–0.717; *p* < 0.001) with *n* = 202 positive cases.

Living alone (OR = 1.62; 95%CI: 0.92–2.88), consuming four or more tablespoons of olive oil per day (OR = 1.66; 95% CI: 0.97–2.83), and less than 2 serves (2 commercially produced pastries) of desserts per week (OR = 2.15; 95%CI: 1.25–3.70) were associated with better mental health. On the other hand, consuming three or more servings/pieces of whole fruit per day (OR = 0.56; 95% CI: 0.31–0.99) was associated with poorer mental health (Table 6).

**Figure 2:**
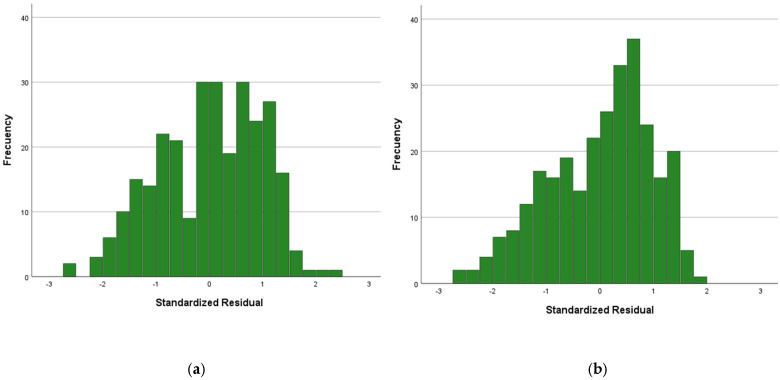
Standardized residual values for Quality-of-life scores (SF-12) regression in (**a**) physical (PCS-12) and (**b**) mental (MCS-12) dimensions.

A sensitivity analysis using robust linear regression was performed using PCS-12 and MCS-12 as continuous outcomes, including the variables used in each model as independent variables. For the PCS-12 variable (Figure 2a) and MCS-12 variable (Figure 2b), no standardized residual values outside the ±3 range were observed (Figure 2a,b), and Cook’s distance was 0.004 (SD 0.13) for both models.

## 4. Discussion

### 4.1. Adherence of MD and Quality of Life

Surprisingly, adherence to the MD was not significantly associated with HRQL nor with its physical and mental components [49]. Contrary to these findings, results from the PREDIMED-Plus study performed on Spanish individuals aged 55–70 years reported that higher adherence to the MD was independently associated with significantly better scores across all dimensions of HRQL measured using the SF-36 questionnaire [50]. Another study performed in Spain with a random sample of the population aged 35–74 years found that the Mediterranean diet score, calculated according to tertile distribution of energy-adjusted food consumption, was significantly associated with a better HRQL measured using the SF-12 questionnaire [24]. One possible explanation for the lack of association between adherence to the MD and HRQL is that 60.7% of participants had good MD adherence, and older adults with lower levels of adherence were underrepresented. This limited variability may have reduced the ability to detect significant associations with HRQL. According to the results obtained in our study, Perez-Tasigchana et al. [26] reported no significant association between MD adherence and better HRQL after two years of follow-up in two independent cohorts of Spanish community-dwelling older adults. However, given that some aspects of the impact of diet on HRQL may not have been reflected by the SF-12 questionnaire, further research using more comprehensive questionnaires specific to HRQL and diet in older adults is needed [51].

### 4.2. Associations Between Physical Component of Quality of Life and Specific Food of MD

Interestingly, we found that the consumption of certain foods, either recommended or limited in the MD, was differentially associated with the good physical and mental components of HRQL. Regarding the physical component of HRQL, older individuals consuming less butter (<1 serving per day) had a higher probability (OR 12.23) of reporting better physical HRQL. A study of Saudi primary health care physicians reported a 90% decrease in HQLR (short version of the World Health Organization HRQL questionnaire) among those using butter and animal fat in cooking [52]. Although direct research on butter consumption and quality-of-life scores is limited, the health impacts of butter intake, such as increased mortality risk, would likely negatively affect an individual’s quality of life [53]. In terms of saturated fat intake, butter consumption had a negative association with the physical functioning domain of QoL in older adults at risk of heart failure [54,55]. High and moderate butter intake also resulted in increases in total cholesterol and LDL cholesterol compared with olive oil consumption and habitual diets [46], thus contributing to cardiovascular alterations that could, in turn, affect the physical component of HRQL. In the logistic model, the low intake of sugar-sweetened beverages was also significant in multivariate analysis (OR 4.19); confirming results of previous studies that have reported associations between sugar-sweetened beverages and reduced HRQL [56,57]. In the explanatory model for optimal physical HRQL, we found that younger participants displayed higher odds of better physical health, which is consistent with the well-known increase in disease burden and increasing mobility problems with ageing and thus with reduced HRQL [5,58,59].

### 4.3. Associations Between Mental Component of Quality of Life and Specific Food of MD

We found that MD recommendations for specific items were significantly associated with a better mental component of HRLQ. Olive oil intake (≥4 servings) was associated with a better mental health score for HRQL. Olive oil, which contains monounsaturated fatty acids (predominantly oleic acid), polyphenols, and vitamins (with vitamin E being the most abundant) [60,61,62] is responsible for many positive health effects, such as a reduction in cardiovascular diseases, neurodegenerative disease, and cancer [52,53]. Within the Moli-sani project in Italy, a population-based cross-sectional study, a positive association was reported between better mental health (HRQL assessed using the SF-36 questionnaire) and a diet enriched with olive oil [21]. Reduced dessert intake (<2 servings) was significantly associated with a better component of HRQL. The relationship between dessert intake and mental HRQL in older individuals is a nuanced topic as it involves multiple dimensions—nutritional, psychological, and social ones [63,64,65]. A possible negative effect of overconsumption of sugar-containing foods like desserts is an increase in blood sugar fluctuations, which can contribute to mood swings, irritability, and fatigue [66,67,68]. Diets high in added sugars have been linked with greater depression risk [65].

The potential beneficial effects of vegetable and fruit consumption on physical and mental health are believed to be due to their high concentrations of antioxidants, polyphenols, vitamins, and omega-3 fatty acids, which are present in these foods and are important in the Mediterranean diet [29,69].

However, and surprisingly, consuming the recommended amount of fruit (>3 servings) was associated with a significant reduction in the mental health component of HRQL. Evidence in the literature suggests that fruit intake, often evaluated together with vegetable consumption, is generally associated with better mental health across various populations, including older adults [70,71]. However, the protective effect of fruit intake appears to be lower than that of vegetables in relation to depressive symptoms [72]. Many older individuals experience reduced appetite at dinner time [73], and they consume more fruit for dinner than the recommended protein portions. It is plausible that those older individuals with poorer mental health cook less and therefore rely more on fruits and dairy products, which require minimal preparation, as their main evening meal, particularly if they live alone. A European study conducted across five countries, including Spain, examined the relationship between good and poor appetite and protein intake in older adults [74] and found that the association between fruit consumption (at different times) and lower protein intake varied according to appetite: fruit consumption at dinner reduced the risk of insufficient protein intake in those with a poor appetite, while fruit consumption at lunch or snack time increased the risk in older adults with a good appetite [74].

Future studies need to evaluate the influence of day-time fruit consumption on protein intake, mental health issues such as depressive symptoms, and social factors [41,66,67], to clarify the apparent discrepancy observed in our study, where lower fruit intake was associated with higher odds of good mental HRQL.

### 4.4. Limitations of the Study

The main limitations of this study are the cross-sectional design, which does not allow for causal inferences. In addition, the use of convenience sampling limits the generalizability of the results, as participants may not fully represent the general population, and therefore the results should be interpreted with caution. Furthermore, the older adults who participated in the study attend activity centers for older adults and are, by definition, more active, healthier, and more socially engaged than the average older adult population, including people with reduced mobility or chronic diseases [75,76,77,78,79]. Therefore, we cannot extrapolate the results to the population that does not attend these Seniors’ centers for Active Ageing, as their characteristics are different. Furthermore, the fact that we did not observe significant differences between women and men could be due to the fact that men are underrepresented in this population, as we have observed in previous studies [36,38]. This study was based exclusively on self-reported data, which may be affected by social desirability bias. Therefore, although our findings identify an association between patterns of consumption of certain MD foods and quality of life in older adults living in the community, they cannot be taken as direct evidence of actual consumption patterns.

## 5. Conclusions

To our knowledge, this is among the first studies to examine associations between individual MD items and HRQL in older adults, considering both physical and mental components separately. In our sample, the physical component of quality of life is not only associated with age (the older you are, the lower your quality of life is on a physical level, as is logical), but eating vegetables and limiting the consumption of butter and sugary drinks is associated with a better quality of life. However, the items in the MD that are associated with better mental health related to quality of life were higher consumption of olive oil and lower consumption of fruit and desserts. These findings highlight the importance of these foods, provide valuable information for personalized dietary interventions, and emphasize the fundamental role of maintaining adherence to the Mediterranean diet and paying attention to the intake of certain foods to mitigate age-related physical and mental health decline and promote overall well-being. Given the complexity of the relationship between diet and health, priority should be given to promoting adherence to Mediterranean dietary patterns throughout life, together with physical activity in older people, in order to preserve functional fitness and quality of life.

Understanding the associations between quality of life and consumption or avoidance of certain foods, which require confirmation in longitudinal studies, could help inform future nutritional interventions aimed at improving both physical and mental health in older adults.

## Figures and Tables

**Table 1:** Mediterranean Diet Adherence Screener (MEDAS) score.

Questions	One Point Is Given for
1. Do you use olive oil as main culinary fat?	using olive oil as the principal source of fat for cooking, or for consumption
2. How much olive oil do you consume in a given day (including oil used for frying, salads, out-of-house meals, etc.)?	4 or more tablespoons of olive oil per day (1 tablespoon = 13.5 g including that used in frying, salads, or meals eaten outside the home, etc.)
3. How many vegetables do you consume per day? (1 serving: 200 g [consider side dishes as half a serving])	2 or more servings of vegetables per day
4. How many fruit units (including natural fruit juices) do you consume per day?	3 or more pieces of fruit per day
5. How many servings of red meat, hamburger, or meat products (ham, sausage, etc.) do you consume per day? (1 serving: 100–150 g)	less than one serving of red meat or sausages per day
6. How many servings of butter, margarine, or cream do you consume per day? (1 serving: 12 g)	less than one serving of animal fat per day
7. How many sweet or carbonated beverages do you drink per day?	less than one cup (1 cup = 100 mL) of sugar-sweetened beverages per day
8. How much wine do you drink per week?	7 or more servings of red wine per week
9. How many servings of legumes do you consume per week? (1 serving: 150 g)	3 or more servings of pulses per week
10. How many servings of fish or shellfish do you consume per week? (1 serving 100–150 g of fish or 4–5 units or 200 g of shellfish)	3 or more servings of fish per week
11. How many times per week do you consume commercial sweets or pastries (not homemade), such as cakes, cookies, biscuits, or custard?	fewer than 2 commercially produced pastries per week
12. How many servings of nuts (including peanuts) do you consume per week? (1 serving 30 g)	3 or more servings of nuts per week
13. Do you preferentially consume chicken, turkey, or rabbit meat instead of veal, pork, hamburger, or sausage?	preference for white meats over red meats
14. How many times per week do you consume vegetables, pasta, rice, or other dishes seasoned with sofrito (sauce made with tomato and onion, leek, or garlic and simmered with olive oil)?	2 or more servings per week of a dish prepared with a traditional sauce of tomatoes, garlic, onion, or leeks sautéed in olive oil

**Table 2:** Characteristics of the sample.

	*n*	%
Age, *Mean (SD); range*	74.97 (5.75)	60–93
*Age groups*		
60–69 years	51	17.9
70–79 years	173	60.9
80 years or over	61	21.4
*Sex*		
Women	250	87.7
Men	35	12.3
Cohabitation		
Alone	104	36.5
With one or more persons	181	63.5
*PCS-12 Physical dimension* ^1^		
Good	82	28.8
Low	203	71.2
*MCS-12 Mental dimension* ^2^		
Good	202	70.9
Low	83	29.1
MEDAS ^3^		
Good diet adherence	106	37.2
Suboptimal diet adherence	179	62.8

^1^ PCS-12 (Physical Score), good condition: more than 50 points; ^2^ MCS-12 (Mental Score): good condition: more than 42 points; ^3^ MEDAS: Mediterranean Diet Adherence Screener: good adherence, 10 points or more.

**Table 3:** Median scores for quality of life and adherence to the Mediterranean diet according to gender, age and cohabitation.

Variables	Total	Sex			Age Groups				Cohabitation		
	Median (IQR)	Median (IQR)			Median (IQR)				Median (IQR)		
		Men	Women	*p* Value	60–69 y	70–79 y	80 y or Over	*p* Value	Alone	With Others	*p* Value
PCS-12 ^1^	42.88	43.72	42.27	0.183	43.12	43.56	37.7	0.582	43.29	42.02	0.92
	(33.61–51.09)	(37.66–51.5)	(32.98–51.06)		(36.59–53.29)	(34.48–53.06)	(30.70–45.72)		(34.11–50.41)	(33.29–51.49)	
MCS-12 ^2^	51.09	55.96	50.65	**0.009**	53.58	50.65	50.91	0.206	51.4	50.65	0.395
	(39.97–57.42)	(44.69–58.74)	(39.10–56.89)		(40.74–58.52)	(39.98–57.24)	(39.39–56.60)		(42.65–57.68)	(38.44–57.24)	
MEDAS ^3^	9 (8–10)	9 (7–10)	9 (8–10)	0.52	9 (7–10)	9 (8–10)	9 (8–10)	0.157	9 (8–10)	9 (8–11)	0.447

^1^ PCS-12 (Physical Score) ranges from 0 to 100, with higher scores indicating better physical health; ^2^ MCS-12 (Mental Score) ranges from 0 to 100, with higher scores indicating better mental health; ^3^ MEDAS: Mediterranean Diet Adherence Screener, with scores ranging from 0 (no adherence) to 14 (best adherence). Values in bold represent significant *p* values.

**Table 4:** Median quality-of-life scores (SF-12) in physical (PCS-12) and mental (MCS-12) dimensions according to items of Mediterranean diet adherence (MEDAS).

		PCS-12 ^1^			MCS-12 ^2^		
MEDAS ITEMS ^3^	Yes/No (*n*)	Yes	No	*p* Value ^U^	Yes	No	*p* Value ^U^
1. Olive oil, yes	267/28	43.12 (33.69–51.32)	36.00 (30.02–43.54)	0.063	51.20 (40.03–57.45)	45.47 (38.24–55.60)	0.500
2. Olive oil ≥ 4	150/135	42.00 (33.16–51.05)	43.36 (34.76–52.22)	0.375	52.73 (43.02–57.98)	49.32 (37.65–56.48)	**0.032**
3. Vegetables ≥ 2	212/73	42.94 (33.59–51.83)	42.88 (33.56–49.74)	0.514	51.60 (40.74–57.78)	49.37 (38.08–56.89)	0.279
4. Fruits ≥ 3	178/107	43.14 (34.14–51.17)	40.46 (32.26–51.06)	0.447	49.44 (38.28–56.60)	53.09 (44.64–87.92)	**0.027**
5. Red meat < 1	211/74	42.86 (32.92–51.94)	43.36 (35.37–50.39)	0.610	50.84 (39.75–57.56)	51.90 (40.74–56.40)	0.752
6. Butter < 1	258/27	43.12 (33.68–52.09)	36.59 (30.38–43.80)	**0.008**	51.20 (39.88–57.40)	48.89 (40.22–57.87)	0.995
7. Sugar-sweetened beverages < 1	254/41	43.12 (33.91–51.97)	37.77 (30.21–46.06)	0.078	50.91 (39.63–57.48)	52.10 (44.03–57.02)	0.780
8. Red wine per week ≥ 7/week	4/281	51.50 (32.56–60.99)	58.42 (32.51–66.73)	0.292	42.86 (36.61–51.06)	50.92 (39.98–57.24)	0.266
9. Legumes ≥ 3	114/171	44.08 (33.69–53.04)	40.98 (33.53–49.83)	0.093	52.05 (40.56–57.92)	50.65 (39.75–56.60)	0.434
10. Fish ≥ 3	113/172	40.98 (30.54–52.08)	43.24 (35.72–51.06)	0.182	48.98 (38.79–55.80)	52.95 (40.76–57.92)	**0.029**
11. Desserts < 2	184/101	43.53 (33.73–53.19)	39.86 (33.19–49.20)	**0.034**	51.93 (42.39–57.73)	49.32 (38.44–56.89)	0.129
12. Nuts ≥ 3	162/123	43.04 (34.14–51.04)	41.66 (33.00–52.40)	0.693	50.50 (40.74–56.96)	52.72 (38.51–57.92)	0.759
13. White meat instead of red meat	252/33	42.66 (33.54–51.05)	43.72 (35.90–52.69)	0.509	51.29 (40.08–56.96)	50.68 (38.89–59.47)	0.885
14. “Sofrito” ≥ 2	190/95	43.27 (33.92–51.13)	40.46 (33.00–50.97)	0.209	50.76 (40.00–56.67)	51.20 (39.27–58.80)	0.476

^1^ PCS-12 (Physical Score) ranges from 0–100; the highest score indicates the best condition; ^2^ MCS-12 (Mental Score) ranges from 0–100; the highest score indicates the best condition; ^3^ MEDAS: Mediterranean Diet Adherence Screener; ^U^: Mann–Whitney U; Values in bold represent significant *p* values.

**Table 5:** Binary logistic regression of quality-of-life score (SF-12) for good physical health (PCS-12).

Good Physical Health PCS ^1^-12	OR	95% CI Inf	95% CI Sup	*p* Value
Age	0.91	0.86	0.97	0.001
MEDAS ^2^ ITEMS				
3. Vegetables ≥ 2 servings	1.89	0.97	3.68	0.061
6. Butter < 1 serving	12.23	1.58	94.93	0.017
7. Sugar-sweetened beverages < 1	4.19	1.19	14.90	0.027
13. White meat instead of red meat	0.46	0.20	1.07	0.072

^1^ PCS-12 Physical Health; ^2^ MEDAS: Mediterranean Diet Adherence Screener.

**Table 6:** Binary logistic regression of quality-of-life score (SF-12) for good mental health (MCS-12).

Good Mental Health MCS ^1^-12	OR	95% CI Inf	95% CI Sup	*p* Value
COHABITATION				
Living alone	1.62	0.92	2.88	0.096
MEDAS ^2^ ITEMS				
2. Olive oil ≥ 4 servings	1.66	0.97	2.83	0.064
4. Fruits ≥ 3 servings	0.56	0.31	0.99	0.049
11. Desserts < 2 servings	2.15	1.25	3.70	0.005

^1^ MCS-12 Mental Health; ^2^ MEDAS: Mediterranean Diet Adherence Screener.

## Data Availability

The data presented in this study are available on request from the corresponding author, because the data are not publicly available due to privacy or ethical restrictions.

## Supplementary Materials

The following supporting information can be downloaded at: https://www.mdpi.com/article/10.3390/nu17203243/s1, Figure S1: PCS-12 Calibration plot; Figure S2: MCS-12 Calibration Plot; Table S1: False Discovery Rate for MEDAS items.
