# Supplementary material for: Association Between Mediterranean Diet Consumption and the Physical and Mental Components of HRQL in Community-Dwelling Older Adults in Valencia
Source: Nutrients. 2025 Oct 15;17(20):3243. doi: 10.3390/nu17203243 (PMC12567089; doi:10.3390/nu17203243)
Supplement: Supplementary file 1 [file nutrients-17-03243-s001.zip › nutrients-3906623-supplementary.pdf]

nutrients

MDPI

Supplementary file

Table S1. False Discovery Rate for MEDAS items.

MEDAS ITEMS[1]	PCS[2]-12	Rank PCS-12	Critical PCS-12	Significant PCS-12	MCS[3]-12	Rank MCS-12	Critical MCS-12	Significant MCS-12
1.Olive oil, yes	0.063	3	0.011	FALSE	0.5	9	0.032	FALSE
2.Olive oil ≥ 4	0.375	9	0.032	FALSE	0.032	3	0.011	FALSE
3. Vegetables ≥ 2	0.514	12	0.043	FALSE	0.279	6	0.021	FALSE
4. Fruits ≥ 3	0.447	10	0.036	FALSE	0.027	1	0.004	FALSE
5. Red meat < 1	0.61	13	0.046	FALSE	0.752	10	0.036	FALSE
6. Butter < 1	0.008	1	0.004	FALSE	0.995	14	0.050	FALSE
7. Sugar-sweetened beverages < 1	0.078	4	0.014	FALSE	0.78	12	0.043	FALSE
8. Red wine per week ≥ 7/week	0.292	8	0.029	FALSE	0.266	5	0.018	FALSE
9.Legumes ≥ 3	0.093	5	0.018	FALSE	0.434	7	0.025	FALSE
10. Fish ≥ 3	0.182	6	0.021	FALSE	0.029	2	0.007	FALSE
11. Desserts < 2	0.034	2	0.007	FALSE	0.129	4	0.014	FALSE
12. Nuts ≥ 3	0.693	14	0.050	FALSE	0.759	11	0.039	FALSE
13. White meat instead of red meat	0.509	11	0.039	FALSE	0.885	13	0.046	FALSE
14. "Sofrito" ≥ 2	0.209	7	0.025	FALSE	0.476	8	0.029	FALSE

[1] MEDAS: Mediterranean Diet Adherence Screener; [2] PCS-12 (Physical Score); [3]MCS-12 (Mental Score).

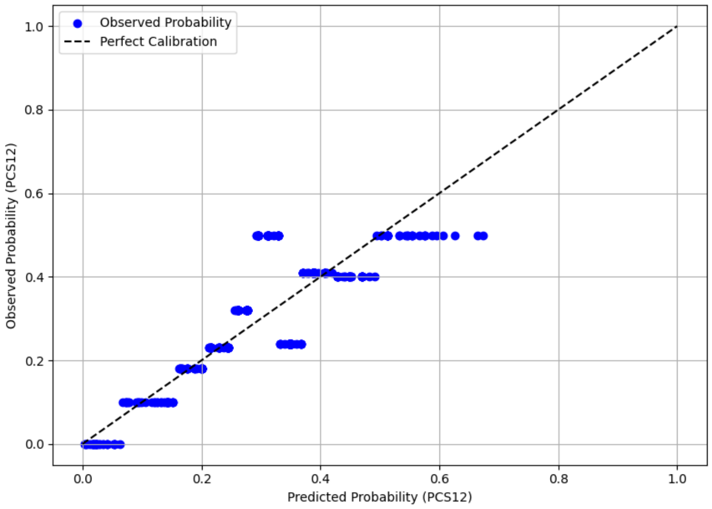

Figure S1. PCS-12 Calibration plot

Legend: PCS-12 (Physical Score)

 https://doi.org/10.3390/xxxxx

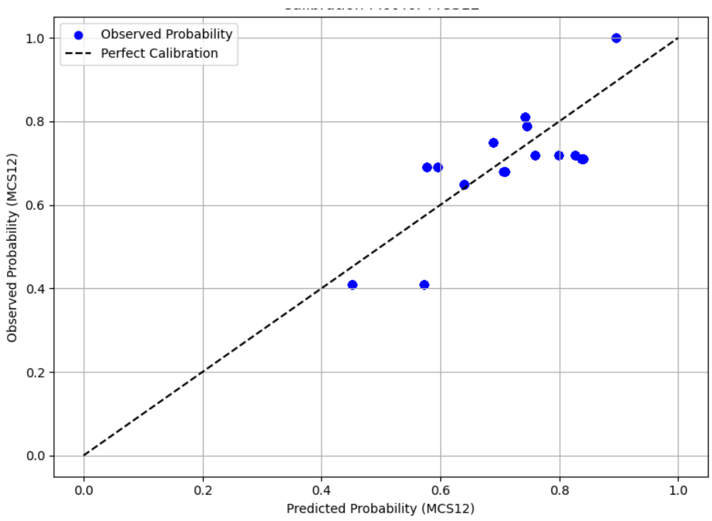

Figure S2. MCS-12 Calibration Plot

Legend: MCS-12 (Mental Score)
